# Cultivating Yet-to-be Cultivated Microbes: The Challenge Continues

**DOI:** 10.1264/jsme2.ME2802rh

**Published:** 2013-06-01

**Authors:** Takashi Narihiro, Yoichi Kamagata

**Affiliations:** 1 Bioproduction Research Institute, National Institute of Advanced Industrial Science and Technology (AIST), Tsukuba Central 6, Tsukuba, Ibaraki 305–8566, Japan; 2 Department of Civil and Environmental Engineering, University of Illinois at Urbana-Champaign, 205 North Mathews Ave, Urbana, IL 61801, USA

When looking into the current situation of microbial ecology, you would realize that the (meta) omics-driven studies associated with next generation sequencing technologies make the headlines in related journals. Currently, isolation and characterization of as-yet-uncultured, but functionally important microorganisms is, at least to a certain extent, being replaced by omics-driven approach without cultivation to decipher their functions. To date, more than 100 microbes have been identified as “*Candidatus*”, that is a provisional status for “well-characterized but as-yet uncultured organisms” (26). Together with this trend, numerous scientists still voice the importance of the cultivation and isolation of microorganisms. Indeed, the pace of proposals on novel species, genus and even higher levels has been incredibly accelerated: *i.e.*, over the past decades, more than 6,000 prokaryotic species have been isolated and characterized on the basis of biochemical, morphological, physiological, and genetic traits (5). However, most of the described organisms are readily cultivable ones, in turn, as-yet-uncultured organisms still remain uncultivable. To fill the gap between the canonical isolation methods and the state-of-the-art technologies that circumvent isolation, developing new approaches to cultivate those as-yet-uncultured organisms in hand is one of the most intriguing challenges in microbial ecology (12, 24).

Significant progress can be highlighted by the description on microorganisms within the class *Anaerolineae* of the phylum *Chloroflexi* (formerly known as *Chloroflexi* subphylum I). Until 2003, the subphylum I within the phylum *Chloroflexi* had not have any cultured representatives whereas it had been well-known as cosmopolitan based on culture-independent molecular analyses. Since the first cultured microorganism was obtained and the novel class *Anaerolineae* was coined, it has gained increased universality. The first cultivated organism named *Anaerolinea thermophila* has been followed by a number of newly isolated organisms within the new genera *Bellilinea*, *Leptolinea*, *Levilinea*, *Longilinea*, *Thermanaerothrix*, and *Ornatilinea*, all of which were within the class *Anaerolineae*, were difficult to isolate but were eventually isolated in pure culture (7, 23, 32). Those were isolated from anaerobic wastewater treatment process, rice paddy soil, and deep terrestrial hot aquifer, and characterized as anaerobic heterotrophic bacteria (32). Moreover, the 16S rRNA gene clones associated with the class *Anaerolineae* were frequently observed in anaerobic wastewater treatment processes, and they may play a role in the degradation of organic compounds such as carbohydrates and amino acids, probably to a great extent, associated with methanogens via interspecies hydrogen transfer (1, 20, 32). In the current issue of Microbes and Environments, Nunoura *et al.* (21) report that *Anaerolineae*-type organisms were predominated in an *in situ* colonization system placed on the shallow submarine hydrothermal vent. They successfully isolate a bacterial strain SW7 and propose the new genus *Thermomarinilinea* with type species *T. lacunofontalis*. Among a number of novel isolates described over the last decade, these organisms are exceptionally well-coordinated on nomenclature basis. Together with genome sequencing of these organisms, we will now know the entity of those organisms that allows us to know exactly who they would be and what they would do, once close relatives are isolated, or omics data that hints at the presence of relatives are obtained.

Methanogenic archaea (methanogens) have a key role in anaerobic ecosystems, where electron accepters other than carbon dioxide (*e.g.*, oxygen, sulfate, and ferric iron) are limited. Previously characterized methanogens have been classified into the orders *Methanosarcinales*, *Methanocellales*, *Methanomicrobiales*, *Methanobacteriales*, *Methanococcales*, and *Methanopyrales* of the phylum *Euryarchaeota* (Fig. 1). Generally, we use laborious culturing techniques with specific apparatuses (*e.g.*, roll tube and agar shake tube) for isolation of the methanogens as well as other obligate anaerobic microbes to eliminate oxygen in the culture medium (8, 21, 30, 31). Such obstacles have led to the difficulty in isolation of obligate anaerobic microorganisms. Nakamura *et al.* (18) developed a simple technique for cultivation of such fastidious anaerobic organisms by using six-well plate and anaerobic gas pack system. They demonstrated the usefulness of this technique to cultivate the methanogens, syntrophic substrate-oxidizing bacteria (syntrophs), and sulfate- or thiosulfate-reducing bacteria. Subsequently, a thermophilic and hydrogenotrophic methanogen, *Methanothermobacter tenebrarum* strain RMAS, was successfully isolated from natural gas field by using this technique (17). More recently, a methanogenic archaeon, *Methanomassiliicoccus luminyensis* strain B10, was isolated from human feces (3). This strain is the first cultured methanogenic representative of the class *Thermoplasmata*. Thereafter, Iino *et al.* (9) report the methanogenic enrichment culture derived from the sludge of an anaerobic digestion process, that contains a novel methanogenic archaeon Kjm51a as a sole archaeal population. Phylogenetic analysis based on the 16S rRNA gene sequences indicates that archaeon Kjm51a is a relative of the *Methanomassiliicoccus luminyensis* but the identity between them is relatively low. According to the phylogenetic and physiological traits of archaeon Kjm51a, they propose “*Candidatus* Methanogranum caenicola” as the provisional taxonomic assignment. Together with *Methanomassiliicoccus luminyensis*, they also propose novel taxa, the family *Methanomassiliicoccaceae* and the order *Methanomassiliicoccales*, for a methanogenic linage of the class *Thermoplasmata* (Fig. 1).

Besides the anaerobic microorganisms, remarkable efforts have been made to cultivate aerobic microorganisms. Fujitani *et al.* (6) develop a bioreactor-based selective culturing technique for the enrichment of *Nitrospira*-type nitrite-oxidizing bacteria (NOB). The affinity to nitrite strikingly affects the growth of dominant NOB, thus nitrite concentration is maintained at a low level to facilitate the specific growth of *Nitrospira*-type NOB and to inhibit the growth of *Nitrobacter*-type NOB. Because the bioreactor-based culturing strategy has the advantage of setting up the stable culture conditions suitable for targeted uncultured microbes, this approach was applied to enrich the yet-to-be cultured organisms: for example, anaerobic ammonium oxidation (anammox) bacteria in coastal sediment (13) and phylogenetically diverse anaerobic microorganisms in subseafloor sediment (10). Tanaka *et al.* (29) successfully isolated a novel aerobic bacterial strain YO-36 from the rhizoplane of an aquatic plant in freshwater environment by using low-nutrient agar medium. Bacterium YO-36 was assigned to the candidate phylum OP10, and proposed as *Armatimonas rosea* of the novel phylum *Armatimonadetes* (28).

In addition, functionally important microorganisms have recently been isolated or enriched: ammonia-oxidizing archaeon (16), aromatic-hydrocarbon-degrading bacteria (11), cellulolytic bacteria (4), chitinolytic bacteria (25), denitrifying bacteria (27), methane-oxidizing bacteria (2), sulfate-reducing bacteria (8), uranium-tolerant bacteria (14), and uric acid-degrading bacteria (30). Clearly, cultivation and (meta) omics approaches should be complimentary. Omics information will give us a clue to the way of isolation of yet-to-be cultivated organisms, and conversely, characterization and genome information of isolates will provide convincing information that would make omics data far more robust. The challenge still continues.

## Figures and Tables

**Fig. 1:**
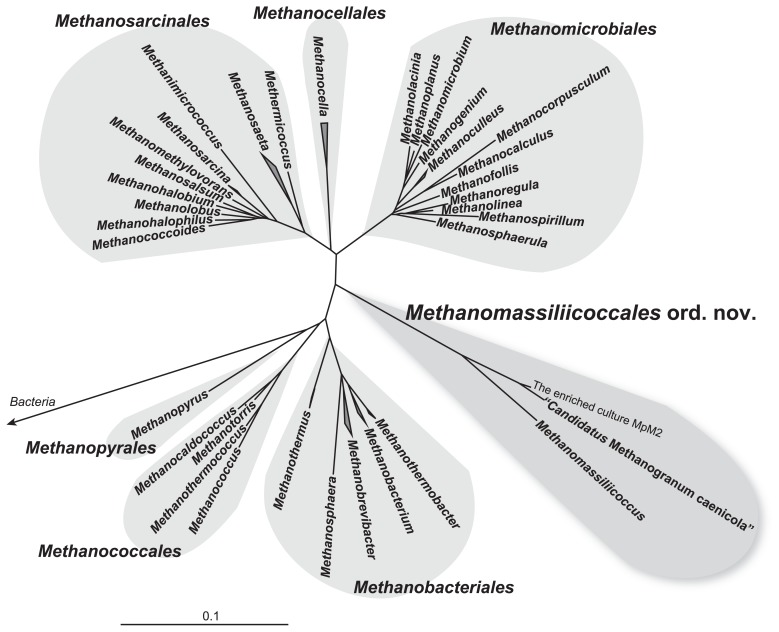
Phylogeny of methanogens. The neighbor-joining tree was constructed on the basis of 16S rRNA gene sequences of previously known methanogens (19) and *Methanomassiliicoccales*-related strains (3, 9, 22) using the ARB software (15). The 16S rRNA gene sequences of the *Thermodesulfobacterium* (AF418169, AF334601, NR_025146, NR_029311) were used as outgroups (not shown). The bar indicates 10% base substitution.

## References

[1] Chen CL, Wu JH, Tseng IC, Liang TM, Liu WT (2009). Characterization of active microbes in a full-scale anaerobic fluidized bed reactor treating phenolic wastewater. Microbes Environ.

[2] Dianou D, Ueno C, Ogiso T, Kimura M, Asakawa S (2012). Diversity of cultivable methane-oxidizing bacteria in microsites of a rice paddy field: investigation by cultivation method and fluorescence *in situ* hybridization (FISH). Microbes Environ.

[3] Dridi B, Fardeau ML, Ollivier B, Raoult D, Drancourt M (2012). *Methanomassiliicoccus luminyensis* gen. nov., sp nov., a methanogenic archaeon isolated from human faeces. Int J Syst Evol Microbiol.

[4] Eida MF, Nagaoka T, Wasaki J, Kouno K (2012). Isolation and characterization of cellulose-decomposing bacteria inhabiting sawdust and coffee residue composts. Microbes Environ.

[5] Euzeby JP (1997). List of bacterial names with standing in nomenclature: A folder available on the Internet. Int J Syst Bacteriol.

[6] Fujitani H, Aoi Y, Tsuneda S (2013). Selective enrichment of two different types of *Nitrospira*-like nitrite-oxidizing bacteria from a wastewater teatment plant. Microbes Environ.

[7] Gregoire P, Fardeau ML, Joseph M, Guasco S, Hamaide F, Biasutti S, Michotey V, Bonin P, Ollivier B (2011). Isolation and characterization of *Thermanaerothrix daxensis* gen. nov., sp nov., a thermophilic anaerobic bacterium pertaining to the phylum “*Chloroflexi*”, isolated from a deep hot aquifer in the Aquitaine Basin. Syst Appl Microbiol.

[8] Higashioka Y, Kojima H, Fukui M (2012). Isolation and characterization of novel sulfate-reducing bacterium capable of anaerobic degradation of *p*-xylene. Microbes Environ.

[9] Iino T, Tamaki H, Tamazawa S, Ueno Y, Ohkuma M, Suzuki K, Igarashi Y, Haruta S (2013). *Candidatus* Methanogranum caenicola: a novel methanogen from the anaerobic digested sludge, and proposal of *Methanomassiliicoccaceae* fam. nov. and *Methanomassiliicoccales* ord. nov., for a methanogenic lineage of the class *Thermoplasmata*. Microbes Environ.

[10] Imachi H, Aoi K, Tasumi E (2011). Cultivation of methanogenic community from subseafloor sediments using a continuous-flow bioreactor. ISME J.

[11] Kaiya S, Utsunomiya S, Suzuki S, Yoshida N, Futamata H, Yamada T, Hiraishi A (2012). Isolation and functional gene analyses of aromatic-hydrocarbon-degrading bacteria from a polychlorinated-dioxin-dechlorinating process. Microbes Environ.

[12] Kamagata Y, Tamaki H (2005). Cultivation of uncultured fastidious microbes. Microbes Environ.

[13] Kindaichi T, Awata T, Suzuki Y, Tanabe K, Hatamoto M, Ozaki N, Ohashi A (2011). Enrichment using an up-flow column reactor and community structure of marine anammox bacteria from coastal sediment. Microbes Environ.

[14] Kumar R, Nongkhlaw M, Acharya C, Joshi SR (2013). Uranium (U)-tolerant bacterial diversity from U ore deposit of Domiasiat in North-East India and Its prospective utilisation in bioremediation. Microbes Environ.

[15] Ludwig W, Strunk O, Westram R (2004). ARB: a software environment for sequence data. Nucleic Acids Res.

[16] Matsutani N, Nakagawa T, Nakamura K, Takahashi R, Yoshihara K, Tokuyama T (2011). Enrichment of a novel marine ammonia-oxidizing archaeon obtained from sand of an eelgrass zone. Microbes Environ.

[17] Nakamura K, Takahashi A, Mori C, Tamaki H, Mochimaru H, Takamizawa K, Kamagata Y (2013). *Methanothermobacter tenebrarum* sp. nov., a hydrogenotrophic, thermophilic methanogen isolated from gas-associated formation water of a natural gas field. Int J Syst Evol Microbiol.

[18] Nakamura K, Tamaki H, Kang MS, Mochimaru H, Lee ST, Kamagata Y (2011). A six-well plate method: less laborious and effective method for cultivation of obligate anaerobic microorganisms. Microbes Environ.

[19] Narihiro T, Sekiguchi Y (2011). Oligonucleotide primers, probes and molecular methods for the environmental monitoring of methanogenic archaea. Microb Biotechnol.

[20] Narihiro T, Terada T, Kikuchi K (2009). Comparative analysis of bacterial and archaeal communities in methanogenic sludge granules from upflow anaerobic sludge blanket reactors treating various food-processing, high-strength organic wastewaters. Microbes Environ.

[21] Nunoura T, Hirai M, Miyazaki M (2013). Isolation and characterization of a thermophilic, obligately anaerobic and heterotrophic marine *Chloroflexi* bacterium from a *Chloroflexi*-dominated microbial community associated with a Japanese shallow hydrothermal system, and proposal for *Thermomarinilinea lacunofontalis* gen. nov., sp. nov. Microbes Environ.

[22] Paul K, Nonoh JO, Mikulski L, Brune A (2012). “*Methanoplasmatales,*” *Thermoplasmatales*-related archaea in termite guts and other environments, are the seventh order of methanogens.”. Appl Environ Microbiol.

[23] Podosokorskaya OA, Bonch-Osmolovskaya EA, Novikov AA, Kolganova TV, Kublanov IV (2013). *Ornatilinea apprima* gen. nov., sp nov., a cellulolytic representative of the class *Anaerolineae*. Int J Syst Evol Microbiol.

[24] Puspita ID, Kamagata Y, Tanaka M, Asano K, Nakatsu CH (2012). Are uncultivated bacteria really uncultivable?. Microbes Environ.

[25] Someya N, Ikeda S, Morohoshi T, Tsujimoto MN, Yoshida T, Sawada H, Ikeda T, Tsuchiya K (2011). Diversity of culturable chitinolytic bacteria from rhizospheres of agronomic plants in Japan. Microbes Environ.

[26] Stackebrandt E, Frederiksen W, Garrity GM (2002). Report of the ad hoc committee for the re-evaluation of the species definition in bacteriology. Int J Syst Evol Microbiol.

[27] Tago K, Ishii S, Nishizawa T, Otsuka S, Senoo K (2011). Phylogenetic and functional diversity of denitrifying bacteria isolated from various rice paddy and rice-soybean rotation fields. Microbes Environ.

[28] Tamaki H, Tanaka Y, Matsuzawa H, Muramatsu M, Meng XY, Hanada S, Mori K, Kamagata Y (2011). Armatimonas rosea gen. nov., sp nov., of a novel bacterial phylum, Armatimonadetes phyl. nov., formally called the candidate phylum OP10. Int J Syst Evol Microbiol.

[29] Tanaka Y, Tamaki H, Matsuzawa H, Nigaya M, Mori K, Kamagata Y (2012). Microbial community analysis in the roots of aquatic plants and isolation of novel microbes including an organism of the candidate phylum OP10. Microbes Environ.

[30] Thong-On A, Suzuki K, Noda S, Inoue J, Kajiwara S, Ohkuma M (2012). Isolation and characterization of anaerobic bacteria for symbiotic recycling of uric acid nitrogen in the gut of various termites. Microbes Environ.

[31] Tsai TL, Liu SM, Lee SC, Chen WJ, Chou SH, Hsu TC, Guo GL, Hwang WS, Wiegel J (2011). Ethanol production efficiency of an anaerobic hemicellulolytic thermophilic bacterium, strain NTOU1, isolated from a marine shallow hydrothermal vent in Taiwan. Microbes Environ.

[32] Yamada T, Sekiguchi Y (2009). Cultivation of uncultured *Chloroflexi* subphyla: significance and ecophysiology of formary uncultured *Chloroflexi*‘subphylum I’ with natural and bio-technological relevance. Microbes Environ.

